# Surgical Ligation of Extrahepatic Shunt under Guidance of Doppler Ultrasound, Portography, and Portal Pressure Monitoring

**DOI:** 10.1155/2012/346759

**Published:** 2012-08-13

**Authors:** Mitsugi Shimoda, Takayuki Shimizu, Keiichi Kubota

**Affiliations:** Second Department of Surgery, Dokkyo University School of Medicine, Mibi, Tochigi 321-0293, Japan

## Abstract

A 54-year-old man with chronic hepatitis C was admitted to our hospital because of a disturbance of consciousness and hyperammonemia. Abdominal angiography revealed a portosystemic shunt between the superior mesenteric vein and inferior vena cava. Endoscopic examination showed no varix. As interventional treatment was unsuccessful, surgical ligation of the shunt was performed. After surgery, portography revealed a huge shunt. Before ligation, the portal pressure, portal flow speed, and volume at the umbilical portion were 24 H_2_O. 5.6 cm/s and 203 ml/min, respectively. Finally the shunt was ligated. The portal flow speed and volume increased for 14 days following surgery and then stabilized. No varices were observed postoperatively. Doppler ultrasound, portography, and portal pressure monitoring can be used to reveal haemodynamic changes in the portal system and justify surgical ligation of portosystemic shunt.

## 1. Introduction

Hepatic encephalopathy is one of the major complications observed in patients with liver cirrhosis.

This encephalopathy is divided into two types: acute and chronic. A portosystemic shunt is one of the causes of chronic encephalopathy with hyperammonaemia. Treatment avenues include medication and interventional or surgical shunt occlusion. Although interventional treatment is used widely, patients may suffer severe liver dysfunction due to a significant increase in the portal pressure after procedure [1]. In this paper, a patient with a large extrahepatic portal-caval shunt is described, paying particular attention to the changes in portal pressure, portal-flow speed, and volume before and after surgical shunt ligation. 

## 2. A Case Report

A 54-year-old man was admitted to our medical center because of a disturbance of consciousness and hyperammonemia. He had had chronic hepatitis C infection for 10 years and had also stuffed from nephropathy and retinopathy caused by diabetes mellitus for 20 years. On admission, his blood chemistry showed elevated levels of serum ammonia (416 *μ*g/mL, normal range: 30–86), creatinine (4.8 mg/dL, normal range: 0.6–1.1), BUN (85 mg/dL, normal range: 7–21), and blood sugar (132 mg/dL, normal range: 55–110). Hepatitis C virus antibody was positive.

**Figure 1:**
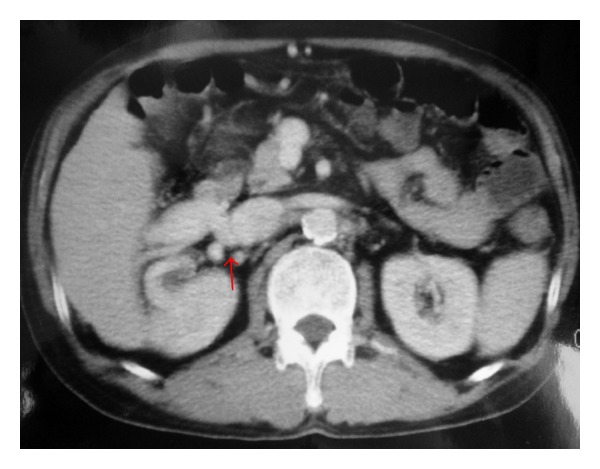
Computed tomographic findings. The portosystemic shunt (arrow) can be seen above the right kidney.

**Figure 2:**
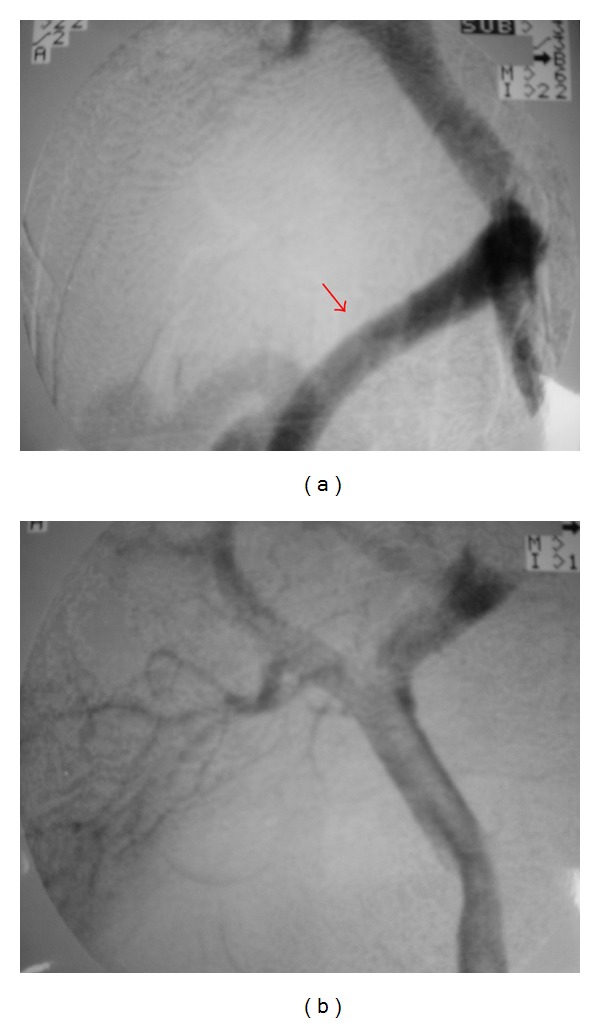
Portographic findings before ligation. Portography through the superior mesenteric artery showed a huge shunt which drained into the inferior vena cava (arrow). Portographic findings after ligation. After shunt occlusion, the huge shunt could not be seen.

**Figure 3:**
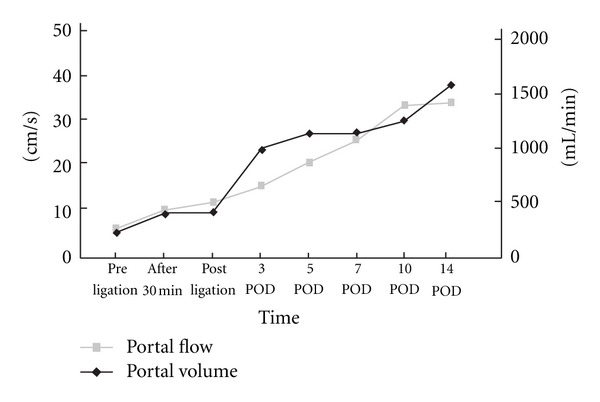
Alteration of portal flow speed and volume. The portal flow speed and volume increased for 14 days postoperatively.

Abdominal computed tomography with contrast material revealed abnormal vessels around the right kidney. There were no ascites and splenomegaly (Figure 1). Endoscopic examination showed no varix. An abdominal angiogram revealed a thick vessel ramifying from the superior mesenteric vein and draining into the inferior vena cava. No other shunts were observed. From the above findings, it was felt that the persistent hyperammonemia and encephalopathy were caused by the portosystemic shunt. Interventional treatment via the femoral vein was attempted to occlude the shunt by a retrograde approach, but this was unsuccessful. The patient underwent surgery on October, 2000. There was a small amount of ascites (approximately 300 mL). At first, a catheter was inserted into the distal superior mesenteric vein via branch of the small intestine and the portal pressure was measured (24.5 cm H_2_O). Portography demonstrated a huge shunt (Figure 2(a)). By mobilizing the duodenum, the whole aspect of the shunt was revealed the huge shunt ramified into three branches. One shunt drained into the right adrenal vein, one drain was into the inferior vena cava, and remaining one was drained into the right renal vein. Doppler ultrasound using Aloka SSD 5000 (Aloka, Tokyo, Japan) showed that a portal flow speed, and volume at umbilical portion were 5.6 cm/s, and 203 mL/min, respectively, before shunt occlusion. After isolation, the shunt was clamped for 30 min and portal pressure, portal flow speed and volume were then measured: these were found to have increased to 33 cm H_2_O, 11.8 cm/s and 389 mL/min, respectively. Finally, the shunts were ligated and removed. After closing the shunt, portosystemic shunt was disappeared on portography (Figure 2(b)). The total amount of bleeding was 610 mL. Postoperatively, liver function tests revealed normal values and the serum ammonia level returned to normal (18 *μ*g/dL) with clear consciousness. The portal flow speed and volume measured on days 3, 5, 7, 10, and 14 postoperatively were not found to have increased significantly after shunt ligation (Figure 3). Although a small amount of ascites was found, it resolved around 2 months after surgery. No varices were observed endoscopically.

## 3. Discussion

 Interventional treatment using a detachable balloon, with a coil or fibrin glue, may be the treatment of choice for occluding a portosystemic shunt [2–5]. Uflacker et al. [6] reported the results of interventional treatment for a portosystemic shunt in five cases. Although four of the five patients had permanent control of encephalopathy, hemodynamic problems including ascites, esophageal varix, and intraperitoneal bleeding were worse after this procedure. When performing interventional therapy, the changes in portal vein pressure before and after treatment can not be monitored. This technique therefore may not always be justified due to the changes in portal vein pressure which may contribute to several complications, including insufficient occlusion of the shunt flow, recurrence of shunt flow, portal vein thrombosis, and appearance of esophageal varix [1, 3, 7].

 When interventional treatment is unsuccessful, surgical ligation of the shunt becomes an alternative approach. Surgical ligation of the shunt has been reported to be associated with high mortality, which might be attributed to a high level of intraoperative bleeding and inadequate selection of patients with poor liver function [8]. Furthermore, surgical treatment is not always possible. In our case, the shunt was present only between the superior mesenteric vein and the inferior vena cava, and therefore surgical ligation was considered possible and was accomplished successfully. Portography should be performed to confirm a portosystemic shunt. This examination should be repeated after shunt occlusion to confirm the completeness of the surgical treatment. The fact that patients who require shunt occlusion usually have liver dysfunction with portal hypertension must also be taken into consideration. A sudden significant increase in portal pressure following shunt occlusion may be associated with postoperative morbidity and mortality, including rupture of the esophageal varix and liver dysfunction. Futagawa [9] stated that patients with Child C liver function, who showed more than a 60% increase in portal pressure after occlusion, had a poor diagnosis. Kato et al. [8] described the creation of a distal splenorenal shunt in addition to closure of a large gastrorenal shunt to prevent varices from rupturing due to the significant increase in portal pressure. In our patients, the shunt was tentatively clamped for 30 min and the portal pressure was measured. The pressure was 33 cm H_2_O with a 34.7% increase and the shunt was subsequently ligated. The percentage increase in portal pressure may be a reliable criterion (<60% increase) for justifying shunt occlusion and the subsequent occurrence of liver dysfunction.

 Hemodynamic changes in the portal vein can be evaluated using Doppler ultrasound. Doppler ultrasound yielded a portal flow speed and volume. In this case, before occlusion, the two values were 5.6 cm/s and 203 mL/min, respectively. After occlusion, they increased to 11.8 cm/s (82%) and 389 mL/min (89%), respectively. Furthermore, several measurements showed that the two parameters increased for 14 days postoperatively and then became stable. This observation suggests that hemodynamic stabilization of the portal vein required a period of 2 weeks after occlusion. The final value was about 1200 mL/min. Three months after surgery, the patient's liver function is good without any varix formation and his quality of life has improved significantly.

 In summary, a portosystemic shunt was successfully occluded by surgery with careful monitoring of portal pressure, portal flow speed, and volume. In order to avoid a sudden increase in portal pressure, careful meticulous monitoring of the patient is appropriate. After occlusion, hemodynamic stabilization of the portal vein required 14 days.

## References

[1] Nagino M, Hayakawa N, Kitagawa S (1992). Interventional embolization with fibrin glue for a large inferior mesenteric-caval shunt. *Surgery*.

[2] Hanna SS, Smith RS, Henderson JM (1981). Reversal of hepatic encephalopathy after occlusion of total portasystemic shunts. *American Journal of Surgery*.

[3] Ozawa M, Hattori K, Ishii S, Kobayashi H, Sakai Y (2000). A patient with acute portal vein thrombosis after percutaneous trans portal oblitalation (PTO) for refractory hepatic encephalopathy. *Acta Hepatologica Japonica*.

[4] Goldman ML, Fajman W, Galambos J (1976). Transjugular obliteration of the gastric coronary vein. *Radiology*.

[5] Kanagawa H, Mima S, Kouyama H (1991). A successfully treated case of fundic varices by retrograde transvenous obliteration with balloon. *Japanese Journal of Gastroenterology*.

[6] Uflacker R, De Silva OEA, Carneiro D’Albuquerque LA, Piske RL, Mourao GS (1987). Chronic portosystemic encephalopathy: embolization of portosystemic shunts. *Radiology*.

[7] Potts JR, Henderson JM, Millikan WJ (1984). Restoration of portal venous perfusion and reversal of encephalopathy by balloon occlusion of portal systemic shunt. *Gastroenterology*.

[8] Kato K, Kondo S, Hirano S (2001). Surgical closure of the gastrorenal shunt with distal splenorenal shunt operation for portosystemic encephalopathy. *Hepato-Gastroenterology*.

[9] Futagawa S, Ohsuga T, Monna T, Tatebe T (1987). Surgical therapy of portal hypertension. *A Textbook of Advanced Clinical Gastroenterology Part II: Liver, Billary Tract, and Pancreas*.

